# Actions of a cancer surveillance technical group based on the perspective of *advocacy* ^*^

**DOI:** 10.1590/1980-220X-REEUSP-2022-0421en

**Published:** 2023-03-27

**Authors:** Franciele Budziareck Neves, Mara Ambrosina de Oliveira Vargas, Laura Cavalcanti de Farias Brehmer, Kely Regina da Luz, Letícia de Oliveira Grespi, Júlia Valéria de Oliveira Vargas Bitencourt

**Affiliations:** 1 Universidade Federal de Santa Catarina, Florianópolis, SC, Brazil.; 2 Hospital de Clínicas de Porto Alegre, Porto Alegre, RS, Brazil.; 3 Universidade Federal da Fronteira Sul, Chapecó, SC, Brazil.

**Keywords:** Health Advocac, Health La, Public Health Nursin, Neoplasms, Defensa de la Salud, Derecho Sanitario, Enfermería en Salud Pública, Neoplasias, Advocacia em saúde, Direito sanitário, Enfermagem em saúde pública, Neoplasias

## Abstract

**Objective:**

To analyze epidemiological surveillance actions for people with cancer based on the perspective of *advocacy*.

**Method:**

Qualitative study, Convergent Care Research type, combined with the framework of health advocacy. It was carried out in the Epidemiological Surveillance of the Health Department of a municipality in the southern region of Brazil.

**Results:**

Eleven health service professionals participated in the study, from June 2020 to July 2021, making up 14 group meetings. Two aspects were discussed: (1) problems involving the management of the work process in the network services, directly influencing user assistance; and (2) issues related to the training management of the professional who works at these services, where the lack of knowledge regarding the legislation leads to serious consequences for the users.

**Conclusion:**

The advocacy strengthened health defense concepts and ideas, mobilizing actions related to cancer, acting as a bridge between the members of the group and the sectors holding power to change circumstances that prevent compliance with public policies and current legislation.

## RESUMO

**Objetivo:**

Analisar as ações de vigilância epidemiológica para pessoas com câncer na perspectiva do *advocacy.*

**Método:**

Estudo qualitativo, do tipo Pesquisa Convergente Assistencial, aliado ao referencial da advocacia em saúde. Realizado na Vigilância Epidemiológica da Secretaria de Saúde de um município na região Sul do Brasil.

**Resultados:**

Participaram 11 profissionais de serviços de saúde, entre junho de 2020 a julho de 2021, perfazendo 14 reuniões do grupo. Discutiram-se dois aspectos: (1) problemas envolvendo a gestão do processo de trabalho nos serviços da rede, que influenciam diretamente na assistência ao usuário; e (2) problemas relacionados à gestão da formação do profissional que atende nestes serviços, onde o desconhecimento relativo às legislações traz consequências graves para os usuários.

**Conclusão:**

O *advocacy* fortaleceu conceitos e concepções de defesa à saúde, mobilizando ações relativas ao câncer, atuando como uma ponte entre os membros do grupo e os setores que detêm poder para modificar circunstâncias que impeçam o cumprimento das políticas públicas e da legislação vigente.

## RESUMEN

**Objetivo:**

Analizar las acciones de vigilancia epidemiológica de personas con cáncer desde una perspectiva de *advocacy*.

**Método:**

Estudio cualitativo, del tipo Investigación Convergente Asistencial, combinado con el referente de *advocacy* en salud. Realizado en la Vigilancia Epidemiológica del Departamento de Salud de un municipio de la región sur de Brasil.

**Resultados:**

Participaron 11 profesionales de los servicios de salud, entre junio de 2020 y julio de 2021, conformando 14 encuentros grupales. Se discutieron dos aspectos: (1) problemas relacionados con la gestión del proceso de trabajo en los servicios de red, que influyen directamente en la atención al usuario; y (2) problemas relacionados con la gestión de la formación del profesional que atiende estos servicios, donde el desconocimiento de la legislación trae graves consecuencias para los usuarios.

**Conclusión:**

La *advocacy* fortaleció conceptos y concepciones de defensa de la salud, movilizando acciones relacionadas con el cáncer, actuando como puente entre los integrantes del grupo y los sectores que detentan el poder para modificar circunstancias que impiden el cumplimiento de las políticas públicas y la legislación vigente.

## INTRODUCTION

Cancer accounts for the highest number of deaths in the world. According to data collected by the World Health Organization (WHO), in 2019, cancer is among the first and second leading causes of mortality before the age of 70 in 112 countries, which clearly demonstrates the considerable reduction in life expectancy of those affected by the disease. Published expectation data for 2020 recorded an incidence of approximately 19 million cases of cancer worldwide, with 10 million deaths. More than 60% of cancer cases are concentrated in the 10 most frequent types, which also account for 70% of all deaths^(1)^.

Faced with alarming statistics, epidemiological surveillance actions with the communities, such as the detection of new cases of neoplasms, recognition of the provision of network services for cancer care, and information on deaths and recoveries, are of fundamental importance. These actions illustrate a specific reality and gather indispensable information to the creation of resolving strategies according to the identified problems, and allow ensuring the person with neoplasia the right to assistance^(2)^.

Surveillance actions, which show data on mortality, incidence and prevalence of cancer in general, manifest frequent innovations, which renew the field with creative ideas, and help to overcome obstacles^(3)^. Post-operative telephone monitoring, following hospital discharge, of cancer patients, although already used, configured a highly effective alternative during the COVID-19 pandemic. This method allows continuity of care, providing education and guidance to patients according to their needs^(4)^. Another effective idea in surveillance actions is the early detection of cervical cancer (CC) through organized screening programs, which rely on Primary Health Care (PHC) to attract women for carrying out the cervix cytopathological examination, and then, if there is a high-grade squamous intraepithelial lesion, refer them to the secondary care network^(5)^.

In this context, when defining the nurse’s role in epidemiological surveillance, it is considered that this professional develops actions of epidemiological investigation, situational diagnosis, planning and implementation of prevention, control and treatment measures, based on information on occurrence and distribution of diseases and diseases in the population. Thus, the epidemiological surveillance nurse, by focusing on reducing the contrast in the health of the population, through public actions and policies, acting in patient defense and providing better results in public health, combines their actions with the advocacy framework in health^(6)^.

*Advocacy* is structured from a set of fundamental stages for its execution, such as: identification of problems that hinder the progress of a cause; search for solutions; promotion of actions aiming at the public sector representatives to rethink public policies for the problem presented^(7)^.

From this perspective, promoting epidemiological surveillance actions for people with cancer, which encourage patient defense, is something new, which opens a gap in knowledge. Therefore, although there are several surveillance actions focusing on people with cancer, the advocacy framework takes a new look at how to apply strategies capable of helping both care professionals and managers in decision-making aiming at the promotion, prevention, treatment, and rehabilitation, with political and social support.

In view of this, the objective of this study was to analyze epidemiological surveillance actions for people with cancer based on the perspective of advocacy.

## METHODS

### Design of Study

This is a study with a qualitative approach, of the Convergent Care Research (CCR) type, which is characterized by improvements with the introduction of innovations in the context of nursing and health care practice, with the innovative change in care practice being the specificity of the CCR and, necessarily, which gives its identity^(8)^. To assert the criteria of qualitative studies and data credibility, the Consolidated Criteria for Reporting Qualitative Research (COREQ) was followed.

The CCR consists of four different phases: conception, instrumentation, scrutiny, and analysis. In the conception and instrumentation phases, the researcher outlines the research problem and defines the research scenario, based on the approximation of the context to practice, that is, the theoretical and methodological support is structured, and the place and means of search are defined. In this case, Epidemiological Surveillance actions for cancer based on the perspective of advocacy and its occurrence connected to the Epidemiological Surveillance Service of the Health Department of a given municipality. In the scrutiny phase, an essential step, the researcher explores his/her capacity as an examiner, extolling effective skills in the investigation of research elements that will be feasible for analysis during instrumentation, and that will be considered for the analysis itself. Finally, in the analysis phase, the researcher, in possession of the syntheses of the scrutiny process, apprehend the data, and makes the synthesis and theorization, ending with the transfer to practice^(8)^.

### Local

The study was carried out at the Epidemiological Surveillance of the Health Department of a municipality in southern Brazil and involved numerous network services related to oncological surveillance actions, such as: High Complexity Oncology Unit (UNACON); Medical Specialties Sector; Outpatient’s Department of a Private University that serves users of the Brazilian Public Health System (SUS); Control, Regulation and Evaluation Service; Municipal Laboratory; and Private Laboratory that analyzes most of the biopsies in the municipality.

### Population and Selection Criteria

The selected population was intentionally defined by the direction of the Epidemiological Surveillance of the municipality surveyed in agreement with the Coordinator of the Epidemiological Oncological Surveillance Group created on December 12, 2019 and with the approval of the Secretary of Health. The professionals were selected to the study based on their notability regarding the purpose of the Oncological Epidemiological Surveillance Group, therefore representing the services of the municipal network considered as allies to surveillance actions.

Professionals from the service who were on vacation or leave were excluded from the study, even if they could be part of the group due to their representativeness during the collection period.

### Data Collection

For data collection, CCR is structured based on the following attributes: dialogicity, expandability, immersibility, and simultaneity^(8)^. This way, CCR attributes associated with the fundamental stages of advocacy were considered: identification of problems that hinder the progress of a cause; search for solutions; action promotion aimed at pressing public sector representatives for them to rethink public policies for the problem presented.

The collection took place from June 18, 2020 to July 29, 2021 during meetings of the municipality’s epidemiological surveillance group, which totaled 14.

The following steps demonstrate the dynamics of data collection, which took place in a space called convergence groups, which precisely gives the nomenclature of the groups organized for the development of a CCR^(8)^.

Step 1 – identification of problems that hinder the progress of a cause and searches for solutions: the thematic basis for discussion in the meetings focused on the problems identified in the investigation of death from cancer. Understanding that based on this variable, the surveillance group would have the key elements to agree on actions aimed at reducing the occurrence of deaths from cancer that could be avoided. Therefore, the group already idealized which actions to be implemented, as well as which network service the action would be directed to. It should be noted that one of the relevant actions was the invitation that the group made to professionals from the services represented, for a clarifying dialogue to reach the best comprehension of the problem. In these cases, professionals had the opportunity to gather information that could be restricted or even lost and these make up a list of real and necessary data for resolving actions.

Step 2 – promotion of actions aimed at pressing the public sector representatives for them to rethink public policies for the problem presented: for this step, the group analyzed the action, verified whether the problem was solved or not. In this case, the main researcher who also coordinated the surveillance group contacted by telephone the professionals called for a meeting and questioned about the actions that were agreed upon and about the resolution, that is, a resumption of the agreements established during the meetings or through letters of recommendation. Letters of recommendation were documents prepared by the members of the group to register and formalize the identification of a problem and the request for resolution. A fundamental agreement that the group agreed upon is that every six months of actions they would issue a report to the Secretary of Health to inform the actions, solutions, and resolutions taken in the period of time and also coming from the group’s workforce.

The convergence groups were characterized for promoting a broad discussion on the focus topic for each meeting, that is, causes of preventable deaths. To conduct this discussion they had a specific case of a given user, exploring the maximum amount of information about this specific case. Based on these discussions, they expanded their knowledge by absorbing theoretical content involving public health legislation and including studies by researchers in the area whose content was relevant for strategic decisions in solving the problem, thus characterizing the CCR stages of dialogicity and expandability. After that, the participants and the researcher entered the immersibility that consisted of a careful analysis of the situation presented, allowing the researcher to both remain in the focus of the research and to act as an agent of the care practice. Simultaneously, possibly the greatest challenge of the CCR, when there is no dominance between research action and practice, the participants and the researcher captured the possible transformations in the context of research and practice and expressed them through the epidemiological surveillance actions promoted by the group.

### Data Analysis

In the analysis phase, data underwent treatment according to four processes: apprehension, synthesis, theorization, and recontextualization that are part of the analysis and interpretation phases^(8,9)^.

Therefore, with the elaboration of this study and the execution of each step, it can be seen that the group was able to perform its activities based on advocacy, strictly aligned with the CCR, as problems hindering the progress of a cause were identified, there was a search for solutions and the promotion of actions trying to pressure public sector representatives to rethink public policies and the work process itself for the problem presented. In addition to articulating the theoretical issues of advocacy, it was also used as a strategy through a set of actions developed to influence the resolution of problems and failures in the health network. The advocacy worked as a bridge between the group members and the sectors that have power to act and change situations considered a problem for the execution of public policies and for the compliance with current legislation on cancer.

### Ethical Aspects

Participants in this study were informed about the research and agreed by signing the Free and Informed Consent Form (FICF). The project was appreciated and approved by the Research Ethics Committee, under Opinion No. 3.946.643 of 2020 and is in compliance with Resolution 466/12 of the National Health Council, which deals with research involving human beings.

## RESULTS

Eleven professionals participated in the study, as described below, according to the service they represented: Epidemiological Surveillance (a nurse responsible for Chronic Noncommunicable Diseases, a nurse responsible for Vital Statistics, a nurse director, and a nursing technician as an executive secretary); High Complexity Oncology Unit (UNACON) (a physiotherapist as coordinator); Specialized Medical Assistance Unit (UAME) (a nurse as coordinator); Health Department (a nurse responsible for Primary Health Care), Outpatient clinic of a Private University that serves users of the Brazilian Public Health System (SUS) (a coordinating nurse); Control, Regulation and Evaluation Service (an administrator appointed by the coordinator); Municipal Laboratory (a coordinating biologist); and a Private Laboratory (a coordinating administrator) which analyzes most of the biopsies in the municipality.

**Figure 1.**
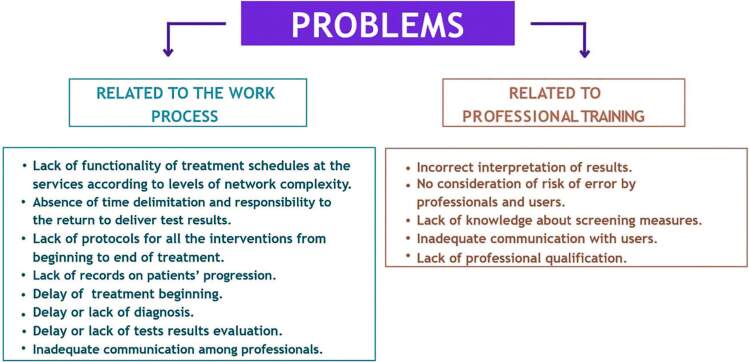
Identification of problems.

The meetings of the convergence groups, whose primary purpose was to discuss cases of preventable cancer deaths and thus propose surveillance actions, allowed the identification of the problems (Figure 1) that could justify these deaths. These problems were categorized for analysis in two dimensions: Management problems related to the Work Process and Management problems related to Vocational Training.

**Figure 2.**
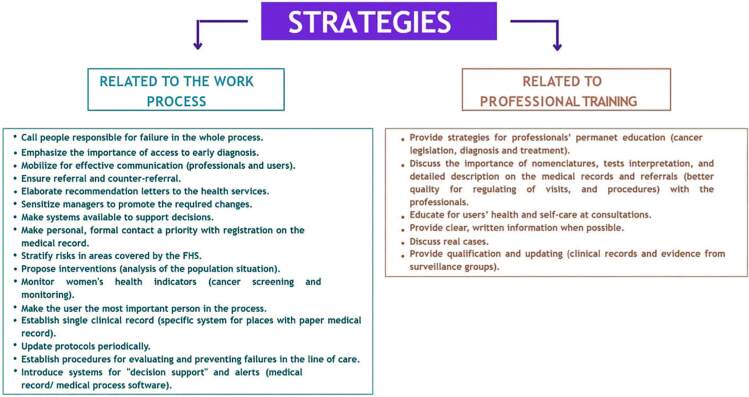
Identification of actions and strategies.

For each of the dimensions of the problems identified by the convergence groups, Epidemiological Surveillance actions were developed to promote improvements (Figure 2).

## DISCUSSION

During the analysis of the main problems identified in the convergence groups meetings and in the surveillance actions for users with cancer, based on the perspective of the advocacy, problems involving the management of the work process in the network services are revealed, as well as in the management of the training workers at these services.

According to Ordinance No. 874, the Ministry of Health establishes the National Policy for the Prevention and Control of Cancer, which has a sequence of regulations aiming at organizing/managing complications, actions and health services, with a view at ensuring full access for the promotion, prevention and recovery for the user with cancer^(10)^. Therefore, knowledge about management and planning, continuing education, and communication ability among the different levels of management are essential for demonstrating problems and actions addressing users with cancer^(11)^.

In this context, the nursing professional exercises a set of management competences, allowing problem-raising, proposition of strategic actions to encourage care improvement, establishment of means, instruments and skills required for this. According to the National Curriculum Guidelines (DCN) leadership, decision-making, administration, and management and permanent education are fundamental skills of nursing for health management and, in this perspective, they play an important role in the management of health service problems and their containment^(12)^.

The Work Process takes place based on its infrastructure, that is, the material resources and physical structure, the instruments used to systematize the work and the professionals involved to support professional practice and allow the operability of a health service. This way, the problems that reveal failures intertwined with the work process imply comprehensive adversities. These are errors that impact the entire system, in their due proportions, and in broader cases, require initiatives for major changes. The work process management directly influences user assistance, especially with regard to continuity of care. Therefore, these problems do not require immediate or short-term actions, but denser attitudes, which make permanent resolutions feasible, as they cover the transformation of health service as whole^(13)^.

Faced with the correlation between the management of the work process and health care, it is noted that the service structure is an elementary component for building care networks, which require results in the face of the growing number of chronic non-communicable diseases^(14)^. Therefore, knowledge about the structure of the network service for people with cancer, assertive measurement and description, based on the reality of problems related to work processes, is fundamental for detecting failures and thus seek solutions that help mitigate them^(15)^. Thus, when obtaining data from an epidemiological surveillance service, as in this study, arising from discussions of real cases, there is a tendency to refer surveillance actions with potential for resolution.

In view of the above, the flaws identified in the service investigated point to problems involving scheduling of consultations and exams, whether for diagnosis or follow-up; the absence of protocols guiding practices in the care trajectory; and communication failures related to records or between professionals.

The delay in scheduling exams and treatments and in releasing results happens for several reasons. In the reality studied, situations related to the transport and delivery of exams were highlighted, and the lack of registration of the exam in the medical record, among other things, is highlighted. These problems involving the logistics of carrying out the tests and treatments hinder the availability of resources and the management of the users’ flow, the information, and the materials^(16)^.

Therefore, the lack of referral and counter-referral also emerged as a problem, even if not explicitly; however, subliminally, communication and organization deficits among services and with users were cited, revealing difficulties in exchanging information in the care and referral network for users, impairing the continuity and fluidity of care. The integration of health services and guidelines according to the function of each service are coherent actions that ensure the mitigation of these problems and the improvement of care^(17)^.

The principle of integrality shall be considered as a means to guarantee the conditions for promotion, prevention, restoration of health, and individuals’ rehabilitation, and all of this should also be the target of surveillance actions. Comprehensive care relies on important changes in practices, both within the organization and articulation of health services and within professional practices that tend to be fragmented and focused on specialization^(18)^. It is believed that integrality considers people as a whole, meeting all their needs. Therefore, the integration of actions and problem solving is vital. However, in this study, non- compliance was observed regarding the principle of integrality, since the actions developed in the services did not work in an integrated way, with the incommunicability between the health network services and among professionals being the indicators to evidence this, which hinders the exercise of care in a broad and holistic way.

The medical record, whether electronic or physical, is a document in which activities related to user care and assistance are recorded. This way, this record shall be carefully done so as not to register wrong information, or even present insufficient elements for the care or its continuity. There are even norms to standardize the language recorded in medical records to facilitate assistance to the service user and which seek to integrate medical records systems^(19)^.

Record failures in the medical record, such as providing incoherent and illegible information, its absence, or even the loss of records/medical records that can be caused by interference in the work process, show an imprecise structure of a given service, which leads to several other problems in the user assistance sequence^(20)^. Therefore, paying attention to the organization of medical records and to the systems themselves, in which they are stored and integrated between the services, as well as to the correct detailing of the records, is a way to ensure health care in its completeness^(19)^.

A study carried out with four municipalities showed that the biomedical model of health care is constantly reinforced by managers, when they exhibit what they consider a priority in health and, consequently, by professionals who tend to adapt to what is demanded by the management. Thus, it is expected that this position centered on valuing care whose nature is healing justifies the distorted priorities of resource allocation and the lack of planning in surveillance^(18)^.

Sequentially, failures related to professional training were observed in the convergence groups, as we know that each member of the multidisciplinary team needs specific improvements. In this case, knowledge and professional commitment before the user with cancer are essential tools that speed up the work process and improve care.

As a result, it could be observed that professionals were unaware of the laws postulating and defending that people suspected of having cancer must be diagnosed and treated within certain periods of time. According to Law No. 13.896, of October 30, 2019, tests to confirm the diagnosis in people whose main hypothesis is a malignant neoplasm must be performed within a maximum period of 30 days^(21)^, and to Law No. 12,732, of November 22, 2012, the patient with cancer already confirmed has the right to undergo the first treatment by the SUS, within sixty days of the diagnosis confirmation^(22)^. Based on these laws, the delay of exams and treatments exceeding these limits constitutes a serious failure of the service, since this delay infringes a user’s fundamental right to receive assistance and health follow-up.

Researchers are interested in debating the lack of information recorded in the SUS databases that justify the delay in responding to the demand required from the user with cancer. It is thought that the delay may involve responses from the institution that performs the treatment as well as responses from users who need care. In this regard, delay and/or misdiagnosis, incorrect referrals and the users’ lack of knowledge about the importance of seeking care since the first symptoms are pointed out as possible causes^(23)^. This discussion corroborates the situations found in the cases investigated by the surveillance group, because even after the publication of the legal rules mentioned, and even with an exponential evolution in diagnostic and therapeutic techniques, contributing to a greater survival and quality of life of this population, there is still a significant number of users affected by the delay in starting treatment.

Studies using the analysis of incidents recorded in incident notification/reporting systems and audits/reviews as their method of investigation showed that the most frequent causes of incidents in Primary Health Care (PHC) were those related to medications (32.5%) and diagnostic error (30.9%)^(24,25)^. Other studies reinforce the previously mentioned findings and identify the probable causes, namely medication-related errors and those at diagnosis are caused by failures in patient records and may be due to pressure for professionals to reduce care time^(26,27)^.

The long waiting time for carrying out diagnostic tests and starting treatment can have serious consequences for users, such as reducing their chances of cure and survival time. In addition, a treatment performed late brings harm to the quality of life, since it requires more aggressive forms of treatment. There is also the increase in public expenses as a result of more expensive and prolonged treatments, as well as in social security costs resulting from absence from work^(28)^.

In view of this, one can reflect that the identification of factors associated with failures detected by the convergence groups in the present study, related to the work process and professional training, allowed directing actions of the epidemiological surveillance group and subsidizing processes of organization, planning and management of services, in the construction of comprehensive and contextually integrated care practices^(29)^ with reality, and theoretical references and professional commitment, which tends to provide effective transformations in health services practices. It is considered that the meetings of the surveillance group are the main actions promoted, since from them emerge great discussions and raising of problems, and from them other actions are promoted as a consequence of the notes made during the discussions, according to the analysis and closing of the cases, after consensus among the group members.

## Limitations

The territorial scope of the study can be perceived as a limitation if we consider that epidemiological surveillance actions for cancer are a worldwide expectation. Therefore, it would be interesting if the study could have been replicated in the same period of time in other municipalities, at least in the state where the initiative with political and social ties was launched. Despite this, in time it is possible to replicate the proposal in other municipal secretaries, improving the scope of the oncological epidemiological surveillance proposal.

## CONCLUSIONS

Developing epidemiological surveillance actions for users with cancer based on the principles of advocacy allied to an action research method allowed researchers and participants to capture the problems involving the causes of deaths from cancer based on the reality of the researched municipality, with regard to the work process of the professionals involved, such as their training for these actions and potential transformations.

The study instigated and inspired the genuine desire of the participants to take on the defense of users precisely because they adopted the advocacy, which strengthened health defense concepts and ideas. In this regard, an in-depth search for causes was triggered and investigated, with digitized data being analyzed, professionals being listened to, policies, legislation and literature on the oncology area being reviewed to obtain maximum subsidies to promote the best surveillance actions in the context studied.

Sustaining the politically proposed strategies regarding the mobilization of management instances and public health managers positions the desired goals at a level of discussions and possible resolutions in a different way. In such a context, to conduct health practices, political and economic power relations are engendered, for which responses are faster and viewed from the perspective of their resoluteness.

Therefore, the research rewarded those involved for mobilizing actions related to cancer, which currently has reached an unwanted and unimagined status by humanity, but which, precisely because it is a serious public health problem, requires proposals that will concretely reduce the dramatic statistics and redesign the scenario and the history of cancer progression in the world.

The creation of epidemiological surveillance groups based on advocacy is encouraged in other municipalities and states in Brazil to enhance the scope of epidemiological surveillance actions for users with cancer.
